# Price variations in food products: A time series dataset for analysis across regions in Italian supermarkets

**DOI:** 10.1016/j.dib.2025.112089

**Published:** 2025-09-29

**Authors:** Daniele Sasso, Luca Bacco, Luigi Palumbo, Juri Marcucci, Niccolò Salvini, Tiziana Laureti, Luca Vollero

**Affiliations:** a Research Unit of Computer Systems and Bioinformatics, Department of Engineering, Università Campus Bio-Medico di Roma, Via Alvaro del Portillo 21, 00128 Rome, Italy; b Research Unit of Intelligent Health Technology for Health and Wellbeing, Department of Engineering, Università Campus Bio-Medico di Roma, Via Alvaro del Portillo 21, 00128 Rome, Italy; c Department of Economics, Engineering, Society and Business Organization, University of Tuscia, Via del Paradiso 47, 01100 Viterbo, Italy; d Directorate General for Economics, Statistics and Research, Bank of Italy, Via Nazionale 91, 00184 Rome, Italy

**Keywords:** Retail prices, Food prices, Supermarkets, Web scraping, Price analysis, Economics

## Abstract

This study presents a comprehensive dataset on retail food prices, specifically covering meat, fruit, and vegetable products, collected through automated web scraping techniques from online supermarket platforms across multiple Italian regions. The dataset spans a period of over two years, from December 2020 to March 2023, and includes structured information on product prices, store locations, and regional variations. Data collection was carried out using Python-based scripts, ensuring automated and consistent extraction of price listings. Supermarkets were geolocated based on their online presence, and products were categorized using the COICOP classification system to facilitate standardized economic analysis.

The dataset enables an in-depth examination of food price dynamics, allowing researchers to investigate regional price disparities, retailer-specific pricing strategies, and temporal price trends across different product categories. By providing granular and time-series data, this resource can support economic studies on inflation, market competition, and consumer purchasing behaviors. Additionally, the dataset can be used for policy-oriented research, aiding in the assessment of food affordability, price volatility, and the impact of external factors such as supply chain disruptions or economic policies. Given its structured nature, the dataset is well-suited for statistical modeling, machine learning applications, and comparative studies on regional price variations within the Italian food retail sector.

Specifications Table

**Table:** 

Subject	Web Scraping / Economics / Retail Pricing / Food Pricing / Computer Science
Specific subject area	Retail price variations of food products across regions in Italy, focusing on fruits, vegetables, and meats.
Type of data	Table, Image, Chart, Graph. Processed, Filtered, Analyzed.
Data collection	Data was collected through web scraping using Python 3.8. Data was gathered from various online supermarkets, with a focus on price listings for fruits, vegetables, and meat products. Data was then categorized based on the COICOP system and processed for regional price comparisons. No manual surveys were conducted, and data was normalized based on the product categories.
Data source location	Data was collected from online supermarkets across various Italian regions, including Lazio, Sicilia, and other provinces.
Data accessibility	Repository name: Variations of Food Prices in Italian Supermarkets Data identification number: 10.5281/zenodo.14927602 Direct URL to data: https://zenodo.org/records/14927602 Instructions The dataset is publicly available on Zenodo and can be accessed via the provided direct URL. No login or special permissions are required. Data can be downloaded in standard formats suitable for analysis.
Related research article	none

## 1 Value of the Data

• **Economic Research Foundation**: This data serves as a resource for analyzing food price trends, inflation, and economic factors influencing consumer behavior, useful for modeling and forecasting inflation.
• **Regional Price Variations**: The dataset provides detailed price time series for food products across Italy, allowing researchers to explore regional price differences and understand local market dynamics.
• **High Granularity of Data**: The dataset includes price information for each unique product, offering high granularity. This detailed level of data enables precise analysis of individual product price fluctuations, enhancing the accuracy of price trend modeling.
• **COICOP Categorization**: The dataset includes categorization based on the COICOP4 and COICOP5 classification system, adhering to the ISTAT nomenclature. This allows for systematic categorization of products, facilitating cross-category and cross-regional comparisons.
• **Additional Use Cases:** The dataset is also suitable for machine learning applications such as food price forecasting, anomaly detection in pricing behavior, or clustering of regional price patterns. Policy makers may leverage the data to evaluate the impact of fiscal or supply chain interventions on food affordability and price volatility. Furthermore, the structured time-series nature of the dataset makes it well-suited for educational purposes, such as classroom exercises on time-series decomposition, seasonality detection, and econometric modeling.

## 2 Background

The retail prices of goods and services, including food products, are influenced by a multitude of factors, as extensively documented in economic literature [[1], [2], [3], [4]]. Among these factors, regional distribution plays a critical role [5,6], leading to significant price variability even within relatively small regions or states. As food prices reflect on the behaviours of the consumers and their nutrition [7,8], understanding these regional price differences is essential for broader market dynamics and the economic implications of pricing strategies. In this context, we constructed a comprehensive dataset aimed at analyzing the retail price variations of meat, fruit, and vegetable products across different regions.

Our motivation for compiling this dataset stems from the need to provide a granular, region-specific analysis of food price dynamics, which has been relatively underexplored in existing studies. While previous research has highlighted the influence of regional distribution on prices, few datasets offer a detailed and continuous view of food price fluctuations over time. Methodologically, we employed web scraping techniques to gather up-to-date pricing data from a diverse range of supermarkets, ensuring that the dataset captures real-time price variations across various geographical locations.

## 3 Data Description

The dataset consists of a single CSV file containing structured tabular data. It does not include separate folders or subfolders. The file can be directly accessed and imported into common data analysis tools. The dataset includes retail prices for meat, fruit, and vegetable products over a period of more than two years, from December 2020 to March 2023. The built database, which is available for further analysis in the supplementary file accompanying this paper, presents data in a tabular format. Table 1 summarizes the columns in the dataset, providing a definition for each. Each row in the table represents a product at a specific point in time, captured on a given day. Alongside the product name and product id, the table includes price, store id and region. This structured format facilitates comprehensive analysis of price variations across different regions and stores.

**Table 1:** Description of the dataset columns, including the specific attributes such as date, price, product identifiers, and the COICOP classification used to categorize products.

Column Name	Definition
*date*	The specific day when the price was recorded.
*price*	The price of the product in the local currency unit, i.e., euros (€).
*product_id*	A unique identifier for the product.
*store_id*	A unique identifier for the store.
*region*	The region where the store is located.
*product*	The name identifying the product.
*COICOP5*	The COICOP5 category of the product, following the ISTAT nomenclature.
*COICOP4*	The COICOP4 category of the product, following the ISTAT nomenclature.

In a preliminary analysis of the dataset, the distribution of products across categories appears slightly imbalanced, with vegetables comprising fewer items (855) compared to fruits (1112) and meats (1171), as shown in Table 2. However, the number of unique products within each COICOP5 category is relatively well-balanced, as illustrated in Fig. 2. Geographically, Lazio emerges as the region with the highest number of unique products, while Sicily exhibits a significantly lower count of both products and stores, as depicted in Fig. 3. When considering the frequency of products, meats clearly dominate, surpassing both fruits and vegetables in terms of prevalence within the dataset (Fig. 4). It is important to note that some products may share the same name but have different product id values. This distinction indicates that, despite having identical commercial designations, they represent different products. Consequently, for accurate analysis, we recommend using the product id as the unique identifier for each product rather than relying solely on the product name.

**Table 2:** Summary statistics of the dataset, showing key metrics for fruit, vegetable, and meat products, such as the number of products per COICOP category, price ranges, and the duration of the longest time series recorded.

Metric	Fruit	Meat	Vegetable
*No of products per COICOP4 category*	1112	1171	855
*No of COICOP5 categories per COICOP4 category*	8	9	7
*No of categories in all regions*	8	9	7
*No of categories in all stores*	7	4	7
*No of products in all regions*	12	6	24
*No of products in all stores*	0	1	2

*Price range*	0.24 ∼ 76.8 €		
*Longest time series*	814 days		

## 4 Experimental Design, Materials and Methods

**Fig. 1:**
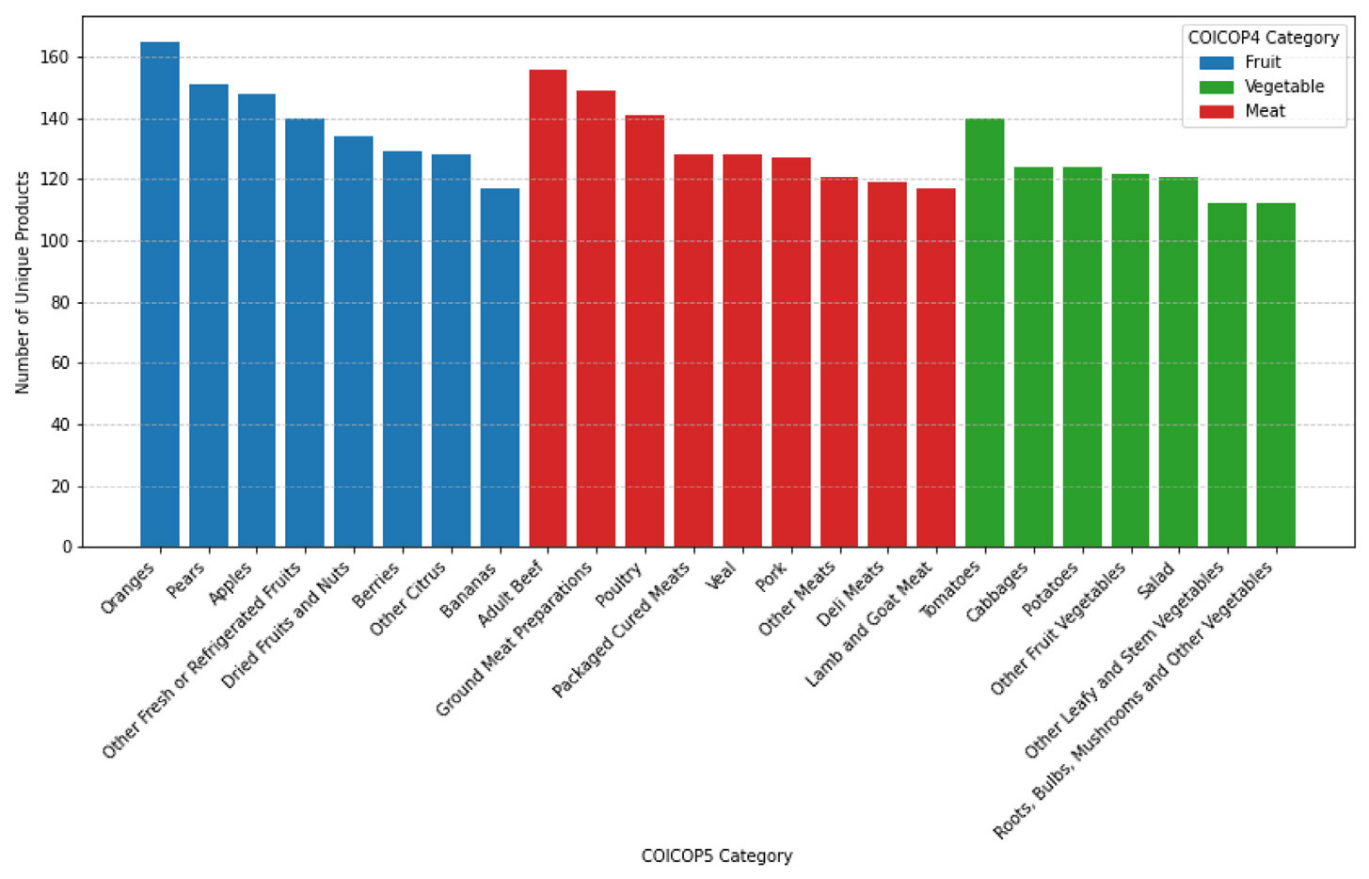
Distribution of unique products across different categories, with fruits represented in blue, vegetables in green, and meats in red. The plot shows the total count of unique products within each category.

The data has been collected through web scraping over a period from December 2020 to March 2023. Prices were obtained from online supermarkets using the “pick up” option for purchase delivery. This method ensures that the price information is accurately linked to the geographical location, maintaining parity between online prices and those applied in physical stores. Each online supermarket was geolocated using GPS coordinates and assigned to a specific municipality. The data collection scripts were scheduled to run automatically daily, allowing the system to continuously gather updated price information from online sources without manual intervention. The data collection covered multiple provinces, including Bergamo, Caserta, Catanzaro, Cosenza, Cremona, Crotone, Frosinone, Latina, Lecco, Milano, Napoli, Palermo, Parma, Perugia, Reggio Calabria, Reggio Emilia, Rieti, Roma, Salerno, and Sondrio. These provinces are distributed across the Italian territory (as shown in Fig. 1) and collectively represent a population of 20,045,962 people, which accounts for ≈ 34 % of the total Italian population. In this study, we focus on a diverse range of food products, particularly those related to fruits, vegetables, and meats. To facilitate a structured approach to data analysis and comparison, we assigned each of the collected products to a specific category based on its characteristics and intended use. To do so, we employed the COICOP nomenclature, a hierarchical classification system used to categorize consumer expenditures, which is essential for economic analysis and statistical reporting, as it allows for a detailed understanding of consumption patterns and expenditure behaviors. At its most detailed level, at the five-digit level, i.e., COICOP5, it provides a granular categorization of goods and services. However, retailers usually do not map each of their products to these categories. That said, the data we collected through web scraping do not present this information. To achieve the COICOP categorization, we first obtained experts’ manual annotations of approximately half of the dataset. Subsequently, we developed a set of rules by leveraging regular expressions and domain-specific knowledge. The dataset was acquired and analyzed using Python 3.8 scripts.

**Fig. 2:**
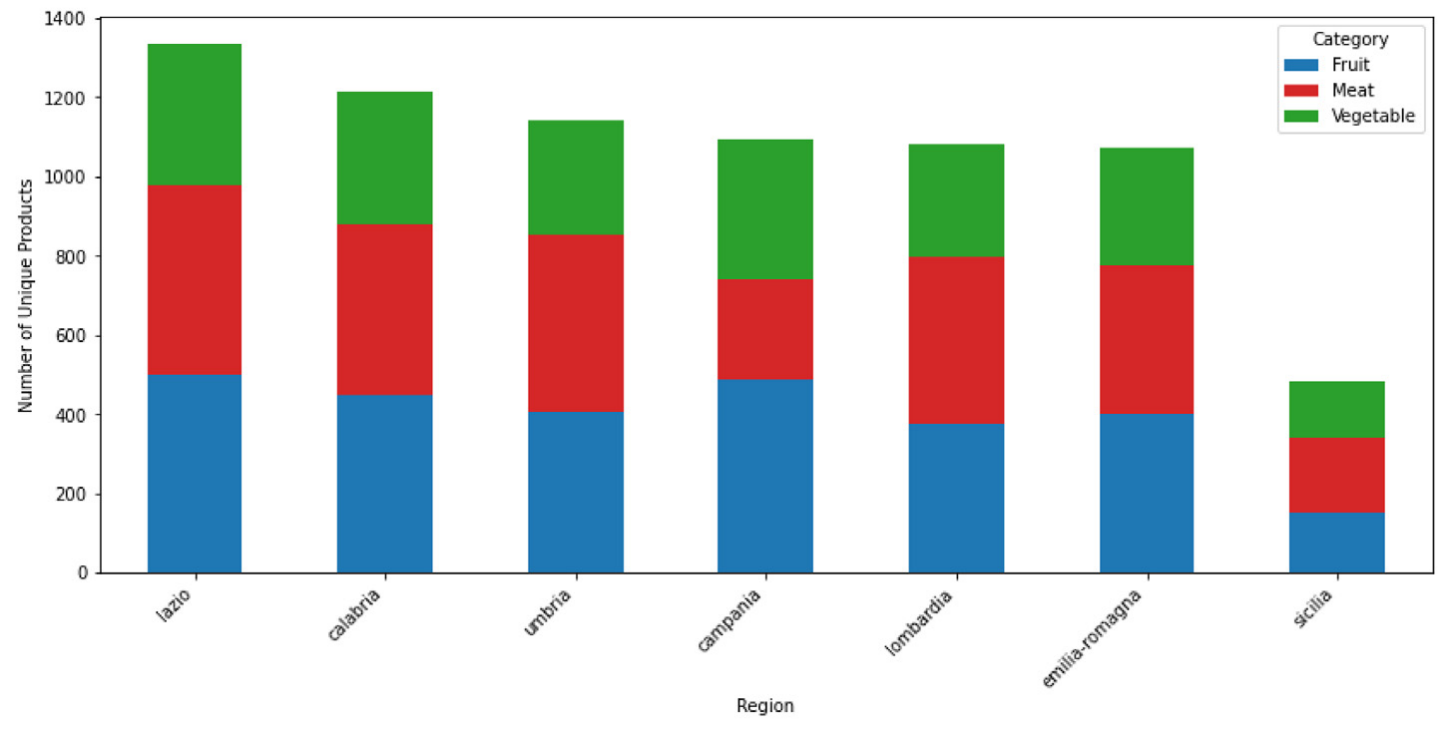
Geographic distribution of products across different regions, with fruits represented in blue, vegetables in green, and meats in red.

**Fig. 3:**
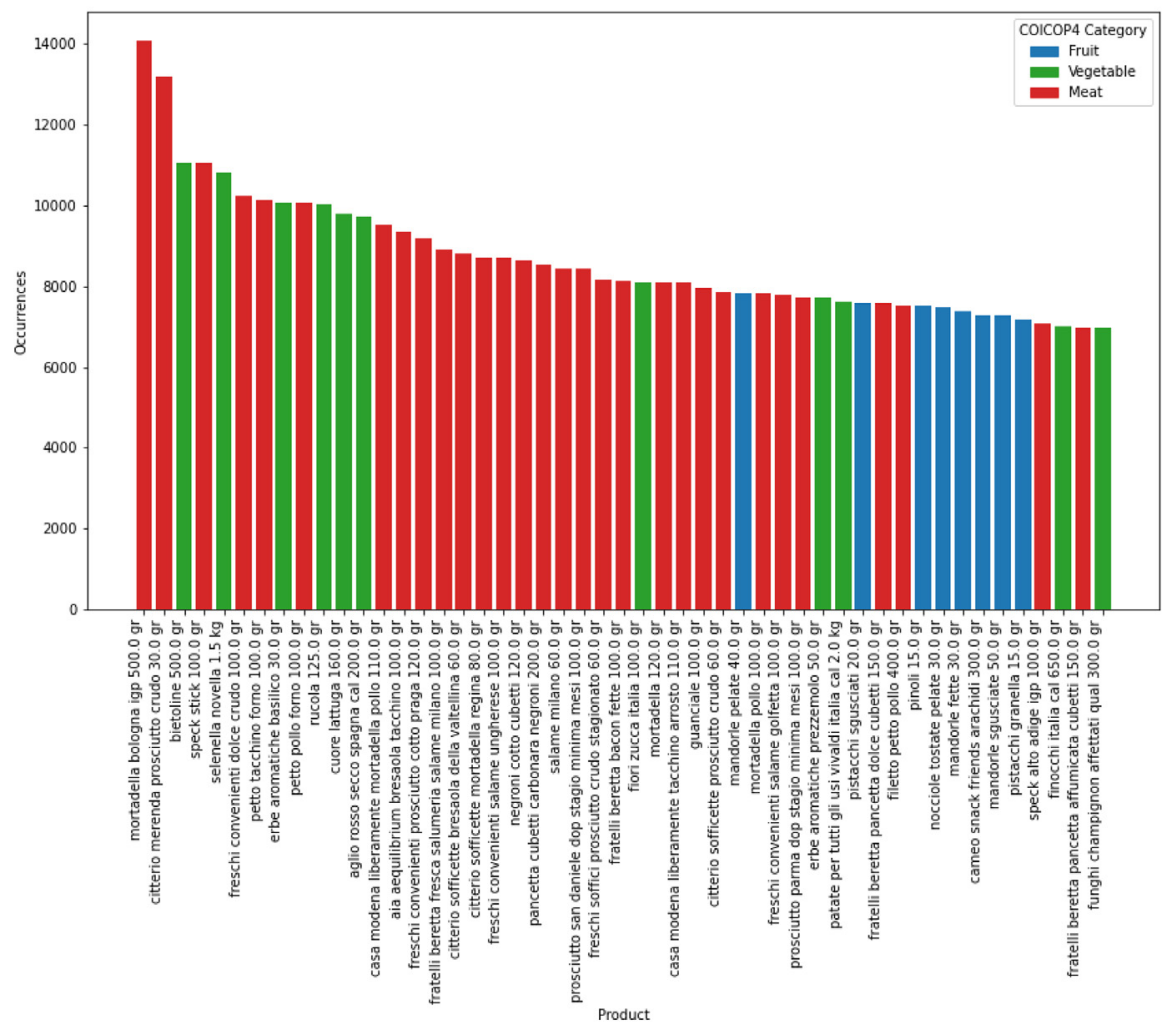
The top 50 most frequently appearing products in the dataset across different product categories, with fruits represented in blue, vegetables in green, and meats in red.

**Fig. 4:**
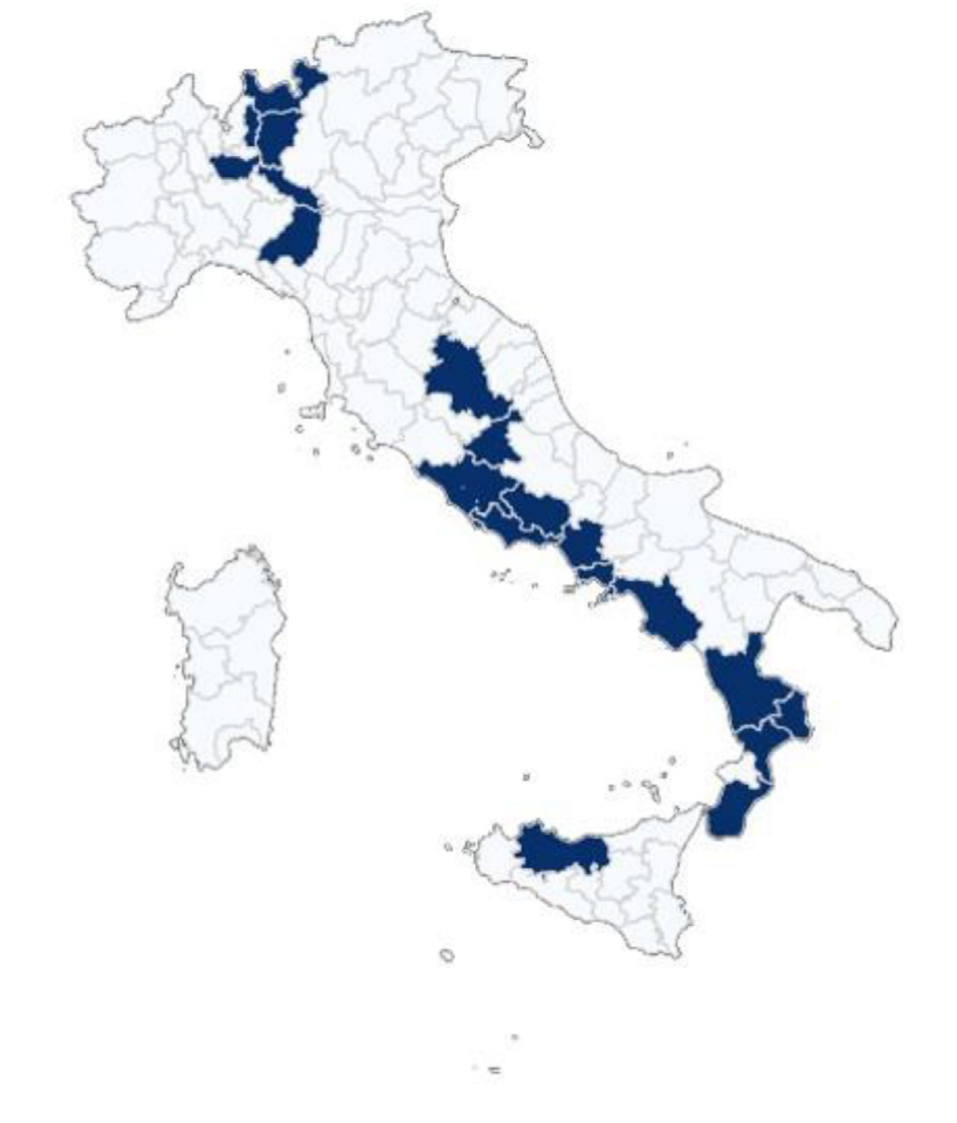
Map of the provinces covered during the data collection routine.

The core steps of the automated data collection process are summarized in the following pseudocode:

*for each supermarket_url in list_of_supermarkets:*

*open website (via requests or Selenium)*

*navigate to target product categories (fruit, vegetables, meat)*

*extract:*

- *product name*
- *price*
- *quantity (from product name)*
- *store metadata (region, store_id)*

*assign product_id if not already existing*

*map product to COICOP5 and COICOP4 categories*

*append to daily dataset with current date*

## Limitations

While the dataset offers valuable insights into regional price variations, several limitations should be noted. First, data collection was restricted to supermarkets with online price listings, which may not reflect pricing in physical stores or smaller, independent retailers. This constraint introduces a potential selection bias toward larger supermarket chains and more urbanized areas, where online shopping services are more commonly available. Consequently, rural zones and small-scale retailers (often lacking an online presence) are underrepresented.

This urban skew may affect the representativeness of the dataset across different regions. For instance, regions such as Sicily display a lower number of unique products and participating stores, which could limit the robustness of regional price comparisons. Users of the dataset should therefore consider these imbalances when analyzing spatial price dynamics or drawing conclusions about broader market behavior.

Additionally, while manual annotations and COICOP-based categorization were applied, some classifications may still contain errors or ambiguities, especially for new or niche products that do not fit neatly into predefined categories. Lastly, the dataset is geographically limited to certain Italian regions, which reduces its ability to support direct cross-country or international price comparisons without additional contextual adjustments.

## Ethics Statement

The authors have read and follow the ethical requirements for publication in Data in Brief and confirming that the current work does not involve human subjects, animal experiments, or any data collected from social media platforms.

## Credit Author Statement

**Daniele Sasso:** Software, Validation, Investigation, Data Curation, Writing - Original draft, Visualization; **Luca Bacco:** Methodology, Software, Formal analysis, Investigation, Visualization, Supervision, Writing - Original draft; **Luigi Palumbo:** Conceptualization, Methodology, Software, Data Curation; **Juri Marcucci:** Visualization, Writing – Review & Editing; **Niccolò Salvini:** Visualization, Writing - Review & Editing; **Tiziana Laureti:** Conceptualization, Project Administration; **Luca Vollero:** Conceptualization, Writing – Review & Editing, Supervision, Project Administration

## Declaration of Generative AI and AI-Assisted Technologies in the Writing Process

During the preparation of this work the authors used ChatGPT in order to enhance the readability of the manuscript, based on text originally written by the authors. After using this tool/service, the authors reviewed and edited the content as needed and take full responsibility for the content of the publication.

## Data Availability

ZenodoVariations of Food Prices in Italian Supermarkets (Original data)
